# Roll Back Malaria: an historical footnote

**DOI:** 10.1186/s12936-018-2582-0

**Published:** 2018-11-19

**Authors:** Sir Richard Feachem

**Affiliations:** Global Health Group, University of California San Francisco, San Francisco, CA 94158 USA

## Abstract

Prompted by the 20th anniversary of Roll Back Malaria, the author recalls hypotheses concerning a major new initiative to control malaria in Africa put forward by WHO AFRO and the World Bank in 1996. These hypotheses, and the reactions to them of a panel of 18 experts, are reviewed and contrasted to the rapid progress and high ambition that characterize the field of malaria today.

## Background

On November 19, 2018, the 20th Anniversary of the Roll Back Malaria Partnership to End Malaria (RBM) will be celebrated in Maputo, Mozambique. During the period leading up to the launch of RBM in New York in 1998, I was the Director for Health, Nutrition and Population at the World Bank and, in that capacity, was involved in the preparation and launch of RBM. The 20th Anniversary prompted me to look back in my files and remind myself about antecedents to the launch of this new partnership.

## The provocation

In June 1996, Dr. Ebrahim Samba, WHO Regional Director for Africa, and I wrote to a panel of international experts as follows.

Dear

Despite many decades of control efforts, malaria remains a leading cause of illness, death, suffering, and poverty in Africa. For reasons that have been much discussed and written about, the traditional armory of preventive approaches is not being fully or consistently used in the most affected areas. New weapons are becoming available and there is much current interest in the effectiveness of impregnated bed nets. In the medium term, an effective malaria vaccine is anticipated and its wide-spread utilization will undoubtedly assist in the control of this disease.

The WHO Regional Office for Africa and the World Bank are interested in exploring a hypothesis (which we attach) with experts in health and development in Africa. WHO and the World Bank are prepared to be advised by Experts on how to intensify malaria control activities in Africa and to seek views on the most appropriate policy and approaches for reduction of malaria burden under the current economic and social environment.

The purpose of this letter is to invite you, a known authority and expert in this field, to express your opinions about the attached hypothesis and related matters. What we seek is five pages of your frank personal thoughts on how it would be best to move forward internationally and nationally on malaria control in Africa. In particular, we would like your review and commentary on the hypothesis. If you agree with it, please tell us why. If you disagree with it, please tell us why and please also propose an alternative hypothesis (which might be that we can do little other than “business as usual”).

We hope you will be willing to contribute your wisdom and experience to this international brainstorming process. We attach a list of the others who have been invited to assist us in the same manner. We will appreciate receiving your thoughts on the subject by June 30, 1996. We will then assemble all the opinions received and come back to you with a proposed next step.

As you will appreciate we are in a very exploratory mode. We do not have a firm position: we do not know where this process will lead us: and we seek the best advice and opinion before making up our minds on these matters.

We send you our personal thanks for taking the time to study this letter and hope that you will be willing to assist us in the manner requested.

With best regards,

Yours sincerely,

Dr. Ebrahim M. Samba Dr. Richard G. A. Feachem

Attached to this letter were six hypotheses that Dr. Samba and I put forward for comment and reaction, reproduced below.

## The hypotheses

The six hypotheses concerning malaria in Africa.

1. Notwithstanding the potential of new tools (such as impregnated bed nets in the short-term and a malaria vaccine in the medium-term) a “business as usual” approach to malaria control in Africa will probably mean that by the year 2050 this disease continues to be a major cause of ill health, death and suffering.
2. There is a potential for a large, long-term, focused initiative to accelerate the pace of malaria reduction.
3. This initiative might operate on a focused geographical basis, selecting initially a small number of areas (perhaps three or four) where rapid progress in malaria control is technically feasible. The initiative would start by establishing effective malaria control in these areas and then move systematically outwards from them to eventually embrace the whole continent.
4. An important purpose of this initiative would be: (i) to strengthen and sustain ongoing high level political and social commitment, both in Africa and among the OECD nations, to the task of malaria control and (ii) To achieve concrete results in reduction of malaria burden by using effectively the tools available (disease management and wide use of personal protection with bed nets) at health services and community levels.
5. The existence of an African malaria initiative would be an incentive to well-focused malaria research investments leading to new products and tools which could be rapidly tested and applied in major ongoing control programmes.
6. Any such initiative would need to take a 30-year time horizon and set a modest goal for the year 2010, a more ambitious goal for the year 2020, and achieve malaria control across Africa by the year 2030.

The panel who received this letter and the hypotheses comprised 18 experts from Australia, Benin, Ethiopia, France, Ghana, Mali, Mozambique, Nigeria, Senegal, South Africa, Spain, Switzerland, UK, USA, Zambia, and Zimbabwe.

## The response

The response from the panelists was enthusiastic. On October 1, 1996, I wrote to Dr. Samba:

“*There was a surprising level of agreement among the panelists and a high level enthusiasm concerning the hypotheses. To summarize the essence of the views of the panel, technical feasibility does not seem to be a stumbling block as long as ongoing evaluation and methodological issues are addressed. Ensuring high*-*level political commitment by African countries, and ensuring that that this is an African based and designed initiative, were deemed vital to success. The participation of OECD countries was widely endorsed as a way to ensure long*-*term policy and financial support, and as a means to tie the initiative closely with a research agenda.*

*The third hypothesis, ‘having the initiative emanate from focused geographical areas’, was much discussed. The panel supported the hypothesis but suggested conditions that would be prerequisites for the success of the initiative. Many experts mentioned that this initiative must be integrated into the existing primary health care system and that its emphasis should be placed at the district level. With this in mind, it was mentioned that significant training at all levels would need to be started as early as possible”.*

Dr. Samba continued to push vigorously for an ambitious new African Malaria Initiative, with support from the World Bank, the American, British and French Governments and other partners. These discussions came together in January 1997 in Dakar, Senegal, at the first meeting of the Multilateral Initiative on Malaria (MIM). The outcome of this first MIM meeting was summarized in a subsequent letter in Nature [1].

## With hindsight

It is interesting to reflect on how we saw the world 20 years ago. I recall that, when drafting the letter and the hypotheses to send to the expert panel, we had a strong sense of being provocative and challenging. Our suggestions came at a time of appalling levels of morbidity and mortality from malaria in Africa and a good deal of fatalism about this situation. Indeed, a number of members of the expert panel took issue with the implication in Hypothesis 2 that malaria in Africa was currently declining.

By today’s standards, with all the remarkable progress that has been made over the past 2 decades, the hypotheses seem tame. In Hypothesis 6, our biggest ambition was to “achieve malaria control across Africa by the year 2030”. There was no mention of elimination. And our proposals sound very cautious in relation to the current deliberations of the *Lancet Commission on malaria eradication* [2] about a malaria-free world within a generation.

The suggestion in Hypothesis 3 concerning the establishment of bridgeheads of malaria control in 3 or 4 areas, which would then be expanded outwards, has clear resonance with the language of Roll Back Malaria and also presaged the concept of *Shrinking the Malaria Map* [3].

The expert panel had interesting and divergent views on the research agenda and new technology. One panelist felt “pretty confident that a vaccine will be developed within 10 years and so the situation in 2050 may not be so grim”. Another said that we are “unlikely to have a suitable malaria vaccine in the medium term, and will face problems of cost and duration of protection”. There was strong endorsement of the tailoring of solutions to local circumstances and that one size does not fit all. There was also consensus on the need for strong Africa-wide and local political commitment and ownership, and that this could not be an effort driven from Geneva or Washington.

Finally, there was wide agreement about the scale and ambition required. In further correspondence dated October 21, 1996, this was summarized as follows:

“*the approach is not to expand and develop work currently in hand, but to think radically about a major new effort. That effort should remain strongly in African hands. The effort would, of course, take full advantage of work already underway through WHO and national governments, but given the initiatives many integrated aspects, it should endeavour to gather broad based support and commitment (donor, national governments, OECD governments) for the launch and to ensure a future for such an initiative. The tone would emphasize that the scale and ambition of this initiative were without precedent.”*

In other words, a broad and bold partnership to end malaria in Africa.
